# Soybean Performance as Affected by Lime and Gypsum Incorporation Through Tillage Versus Surface Application in Pasture-to-Cropland Conversion Areas in Southeast Brazil

**DOI:** 10.3390/plants15081178

**Published:** 2026-04-10

**Authors:** Pascoal Pereira Rodrigues, Josimar Nogueira Batista, Roni Fernandes Guareschi, Claudia Pozzi Jantalia, Bruno José Rodrigues Alves, Segundo Urquiaga, Erica Souto Abreu Lima, Benedito Fernandes de Souza Filho, Jerri Edson Zilli

**Affiliations:** 1 Agronomy Department, Universidade Federal Rural do Rio de Janeiro, Seropédica 23891-000, RJ, Brazil; pascoalrural@gmail.com (P.P.R.); guareschiecotarelli@hotmail.com (R.F.G.); ericaabreulima@gmail.com (E.S.A.L.); 2 Vegetal Production Department, Universidade Federal Rural do Rio de Janeiro, Campus Campos dos Goytacazes, Campos dos Goytacazes 28022-560, RJ, Brazil; josimarbatista@ufrrj.br; 3 Embrapa Solos, Rio de Janeiro 22460-000, RJ, Brazil; 4 Embrapa Agrobiologia, Seropédica 23891-000, RJ, Brazil; bruno.alves@embrapa.br (B.J.R.A.); segundo.urquiaga@embrapa.br (S.U.); 5 Empresa de Pesquisa Agropecuária do Estado do Rio de Janeiro (PESAGRO-RIO), Avenida Francisco Lamêgo, 134, Jardim Carioca, Campos dos Goytacazes 28080-000, RJ, Brazil; beneditopesagro@yahoo.com.br

**Keywords:** soil fertility, soybean productivity, lime and gypsum incorporation, biological nitrogen fixation, soil recovery

## Abstract

Lime and gypsum are widely used to correct soil acidity and improve grain yields in Brazilian agricultural systems. However, limited information is available on their effectiveness and application practices in degraded sandy soils typical of older agricultural frontiers, such as those in Rio de Janeiro State. This study evaluated the effects of surface application versus the incorporation of lime and gypsum into the soil through tillage operations on soil chemical properties, nodulation, and grain yield of soybean cultivars grown in low-fertility Fluvisols. The experiment was conducted during the 2021/2022 growing season in Campos dos Goytacazes, Rio de Janeiro, using a strip-plot design with four soybean cultivars and two soil amendment placement strategies: surface application without tillage and incorporation through tillage. Soil chemical attributes, nodulation, nutrient uptake, and yield components were assessed. Incorporated application significantly increased soil pH, reduced Al^3+^ toxicity, and enhanced Ca^2+^, Mg^2+^, P, and K^+^ availability compared to surface application. Nodulation responses varied among cultivars, with incorporated treatments promoting up to 40% greater nodule biomass. Although primary root length was not affected, incorporation stimulated secondary root development and nutrient uptake, leading to approximately 50% higher pod number and grain yield. Overall, incorporating lime and gypsum through soil tillage was more effective than surface application in improving soil fertility, enhancing nodulation, and increasing soybean productivity under the conditions evaluated in this study. These findings suggest that lime and gypsum incorporation can represent an important management strategy for improving soybean production in degraded sandy soils.

## 1. Introduction

Historically, sugarcane (*Saccharum* spp.) cultivation in Brazil was concentrated in regions near ports, serving as one of the country’s first globally traded commodities. Major production hubs included Rio de Janeiro, São Paulo, and northeastern states such as Alagoas and Pernambuco. Over time, sugarcane expansion shifted to coastal regions of other Brazilian states due to agronomic, economic, and environmental drivers [1]. In areas such as northern Rio de Janeiro, former sugarcane fields were frequently converted to pasture, altering regional land use dynamics [2].

Currently, Brazil ranks as the world’s leading producer of soybean (*Glycine max* L. Merrill), with more than 40 million hectares under cultivation and an estimated production of 124 million tons in the 2023/2024 growing season [3]. Soybean is cultivated across diverse agroecosystems, from the southernmost state under subtropical conditions to the northernmost under a tropical climate, and its introduction has increasingly been adopted as a strategy for rehabilitating degraded pasturelands. This transition enhances soil fertility and provides a market-viable alternative to traditional agricultural systems. In this context, soybean cultivation has recently emerged as a promising land-use strategy in Rio de Janeiro, particularly in areas historically dominated by sugarcane and pasture [4].

Soil, topographic, and land use assessments have identified approximately 320,000 hectares with potential for grain production in northern Rio de Janeiro. Approximately 70% of this area comprises Ferralsols (FR) and Acrisols/Alisols (AC/AL) (locally referred to as “tabuleiro” soils), while the remaining 30% consists of Cambisols (CM), Fluvisols (FL), and Gleysols (GL) (“baixada” soils) [4]. Local studies indicate that soil degradation in this region is largely associated with historical land use practices, including inadequate tillage, unsustainable pasture management, and limited soil conservation measures [2]. These factors have contributed to soil acidification, nutrient depletion, and structural deterioration, particularly in sloping landscapes [5].

The conversion of pasture to cropland presents significant agronomic challenges, particularly with respect to soil acidity, nutrient dynamics, and physical properties. Soil amendment strategies, including the application of lime and gypsum, have demonstrated potential for improving soil chemical and physical conditions, enhancing microbial activity, and optimizing crop performance [6,7]. Liming effectively neutralizes soil acidity by raising pH and reducing aluminum (Al^3+^) toxicity, while gypsum enhances calcium availability in subsurface layers and promotes leaching of toxic Al deeper into the profile, collectively improving root growth in acidic soils [8].

Furthermore, recent studies show that the use of gypsum has extended to other soil regions in Brazil as a strategy to improve subsoil fertility [9]. However, despite this progress, there is still a lack of site-specific and process-oriented data on how distinct lime and gypsum management strategies—particularly surface application versus incorporation—modify the vertical distribution of soil chemical attributes and influence soybean establishment in degraded sandy soils. These soils are characterized by low cation exchange capacity, limited buffering capacity, subsoil acidity, and high susceptibility to nutrient leaching under irregular rainfall and high temperatures. Unlike the highly weathered clayey soils of the Brazilian Cerrado, sandy soils present faster chemical dynamics and reduced residual effects of amendments, which may alter the effectiveness of conventional liming and gypsum recommendations. This knowledge gap is especially critical in regions undergoing conversion from long-term degraded pastures to soybean cultivation, where soil correction strategies must simultaneously mitigate subsoil acidity, improve nutrient availability, and support early root development under climatic constraints.

Soybean cultivation relies heavily on biological nitrogen fixation (BNF) when inoculated with efficient *Bradyrhizobium* spp. strains, meeting up to 90% of the plant’s nitrogen demand through symbiosis [10]. The critical role of BNF in Brazilian soybean production has been extensively documented [11,12,13].

Correction of soil acidity through liming is essential for optimizing soybean productivity. High soil acidity (low pH) restricts plant growth by increasing aluminum solubility and reducing the availability of essential nutrients. Liming neutralizes these effects by raising soil pH and supplying calcium and magnesium, which enhances root development and nutrient uptake efficiency [14]. Traditionally, lime is incorporated into the soil via plowing and harrowing [15]; however, these practices can disrupt soil aggregates, reduce porosity, and exacerbate erosion risks.

With the widespread adoption of no-tillage systems in Brazil, surface liming has gained popularity as a strategy for preserving soil structure while minimizing labor costs [16]. However, its effectiveness remains debated due to the slow vertical movement of lime within the soil profile, potentially limiting its impact on subsurface acidity [17,18]. In water-scarce regions, deeper incorporation of lime may provide more rapid and uniform soil amelioration. Additionally, co-application of gypsum has been recommended to enhance calcium and sulfur availability in deeper soil layers, promote root elongation, and improve overall plant performance under acidic conditions [19].

The integration of novel soil management technologies alongside conventional soil amendments has the potential to mitigate constraints related to chemical and physical soil properties, facilitating optimal crop development and maximizing agronomic input efficiency [20,21]. Within this framework, key research questions arise regarding the agronomic viability of transitioning pasturelands to soybean cultivation in low-fertility soils: (i) How do soybean plants respond to lime and gypsum applied at the soil surface compared with their incorporation through tillage? (ii) Do different soybean cultivars exhibit distinct nodulation and nitrogen accumulation responses under such conditions?

This study aimed to address these questions by evaluating the agronomic performance and nodulation capacity of modern soybean cultivars grown in acidic soils under different lime and gypsum placement strategies in an area with a historical land use trajectory involving sugarcane followed by pasture. Lime and gypsum were always applied together in this study; therefore, the effects discussed refer to the combined effects of these amendments and their placement in the soil profile (surface application versus incorporation through tillage) rather than to the isolated effects of each amendment.

## 2. Results

### 2.1. Soil Fertility

The soil fertility variables studied are shown in Table 1. The soil chemical analysis revealed no significant interaction between soil amendment placement (i.e., surface application vs. incorporation through tillage) and soil sampling depths for pH and Al^3+^ concentrations. The incorporation of lime and gypsum was more effective than surface application, resulting in an average increase of 0.5 units in soil pH compared to surface treatment. Al^3+^ concentrations were consistently higher at all depths under surface application than with incorporation.

There was no significant interaction between soil amendment placements and sampling depths for Ca^2+^ and Mg^2+^ levels. The highest levels were observed in the top 10 cm, which gradually decreased to lower depths. Higher levels of Ca^2+^ and Mg^2+^ were observed in the area with incorporated lime than in the area with surface application.

There was a significant interaction between the soil amendment placements and depth of the sampled soil regarding available P. The area with incorporated lime and gypsum consistently presented higher values than those with surface application at all evaluated depths. Overall, P levels were significantly higher in the top 10 cm than at other depths, regardless of the soil amendment placements. Notably, exchangeable *p* values (Mehlich 1) were approximately 15, 60 and 330% higher for all sampled soil layers (0–10; 10–20 and 20–40 cm, respectively) in the incorporation treatment compared to that in the surface application.

The overall trend for K^+^ was similar to that observed for P. Higher average values of K^+^ were recorded at all depths in the area with incorporation of lime and gypsum than in the area with surface application. K^+^ levels decreased with increasing depth in the area with surface application. However, there was no difference in K^+^ levels between the 10–20 cm and 20–40 cm layers in the area with incorporation, although they were lower than that of the surface layer (Table 1).

The area with incorporated lime and gypsum exhibited lower average soil organic matter (SOM) values at all depths, with a significant interaction between lime and gypsum application methods and soil depth, decreasing the SOM content. There was no significant difference in the SOM percentage between the 0–10 cm and 10–20 cm layers in this area; however, a significant lower value was found in the 20–40 cm depth. In the area with surface application, the highest SOM content was observed in the 0–10 cm layer, which differed significantly from other depths and, no significant differences were found between 10–20 and 20–40 cm layers. The overall trend was a gradient of SOM with increasing depth.

There was no significant interaction between the lime and gypsum application methods and sampling depth for the exchangeable bases (S) and base saturation (V%). The highest S and V% values were observed in the first 10 cm depth. The area with incorporation showed higher average values of S and V% at all depths compared to the surface application, with a decreasing gradient of S and V% with depth.

A significant interaction between the liming and gypsum methods and sampling depths was also identified for Al^3+^ saturation (m%). The average m% values were lower in the areas with incorporation at all depths. In this area, the m% values were similar between the 0–10 cm and 10–20 cm layers and between the 10–20 cm and 20–40 cm layers. A gradual increase in m% with depth was observed for the surface application, which was higher below the first 10 cm.

The cation exchange capacity (CEC) of the soil showed no significant interaction between the soil amendment placement and sampling depth. However, the incorporation of lime and gypsum resulted in lower average CEC values at all depths, which were significantly different from the surface application. No significant differences in CEC were observed between the depths for either soil amendment placement.

### 2.2. Nodulation, Root Development, and Shoot Dry Matter of Soybean Plants

No interactions between the soybean cultivars and soil amendment placement were observed for plant nodulation. However, significant differences in nodulation were observed between soil amendment placement and soybean cultivars (Table 2). Cultivar M 5917 IPRO had the highest average values for both nodule number and mass, which differed significantly from those of the other cultivars (Table 2). The nodule mass was higher when incorporation was carried out compared to the surface application, although there was no significant difference in the nodule number. No interaction between factors was observed for primary root length, and there were no significant differences between the cultivars or soil amendment placement (Table 2). However, a significant interaction was observed for the number of secondary roots, with the highest average values observed in areas where lime and gypsum were incorporated (Table 2).

Regarding root dry mass, cultivar M 5917 IPRO had the highest average value, followed by BRS 5980 IPRO and 95R95 IPRO, which did not differ significantly from each other or from cultivar BRS 7981 IPRO. Additionally, no significant difference in root dry mass was observed between the soil amendment placement, and there was no interaction between the soil amendment placement and cultivars (Table 2).

The highest values of shoot dry matter at the R1 stage were recorded for the M 5917 IPRO, BRS 5980 IPRO, 95R95 IPRO, and BRS 7981 IPRO cultivars. Overall, M 5917 IPRO produced the largest amount of dry matter, followed by BRS 5980 IPRO, BRS 7981 IPRO, and 95R95 IPRO (Table 2). However, the incorporation of lime and gypsum resulted in the highest shoot dry matter, independent of the soybean cultivar.

### 2.3. Nutrient Accumulation in the Aerial Parts of Soybean

Soybean shoot dry matter analyses at the R6 stage showed no interaction between soil amendment placement or cultivars (Table 3), indicating similar behavior across soybean cultivars under both soil amendment placement. Consistent with the shoot biomass trend, the cultivar M 5917 IPRO generally accumulated more Ca^2+^, Mg^2+^, K^+^, and N than the other cultivars. Cultivars BRS 5980 IPRO and 95R95 IPRO displayed intermediate nutrient accumulation, whereas BRS 7981 IPRO accumulated the least nutrients, except for P (Table 3). N accumulation in M 5917 IPRO was nearly 50% higher than that in BRS 7981 IPRO (Table 3). Regarding the comparison soil amendment placement, significantly higher values were observed in the incorporation area, with increases of 38%, 28%, 24%, 19%, and 33% for Ca^2+^, Mg^2+^, K^+^, P, and N, respectively.

### 2.4. Production Components and Grain Productivity of Soybeans

**Figure 1:**
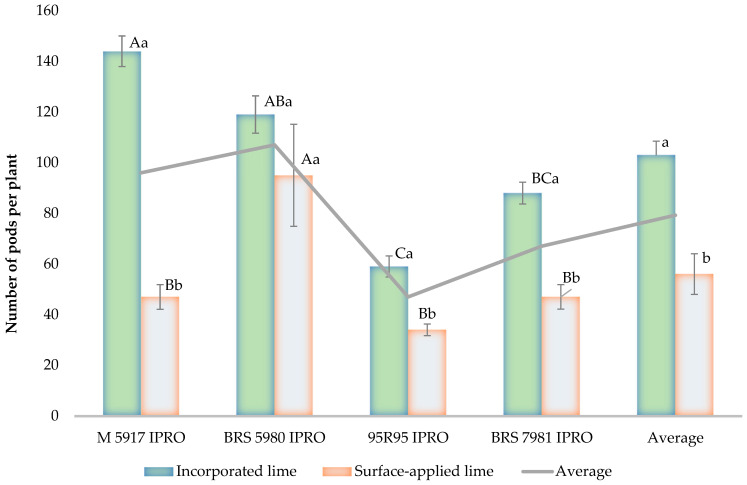
Number of pods per soybean plant in an experiment comparing soil amendment placement in Campos dos Goytacazes-RJ, Brazil, during the 2021/2022 season. Identical uppercase letters indicate statistically similar means between cultivars, while lowercase letters denote similarities between soil amendment placement according to the Tukey test at a 5% probability level. Data were log-transformed (log (y)). For the strip-plot design, after transformation, the coefficients of variation were CV_1_ = 3.8%, CV_2_ = 4.6%, and CV_3_ = 5.9%.

The M 5917 IPRO and BRS 5980 IPRO cultivars had the highest number of pods per plant, followed by BRS 7981 IPRO, which outperformed 95R95 IPRO (Figure 1). The M 5917 IPRO cultivar had fewer pods than the BRS 5980 IPRO in the treatment with surface-applied lime and gypsum (Figure 1). In fact, there was an interaction between soil amendment placement and cultivars, with the area where the incorporation was carried out showing approximately 50% more pods per plant than the surface-applied lime (Figure 1).

**Figure 2:**
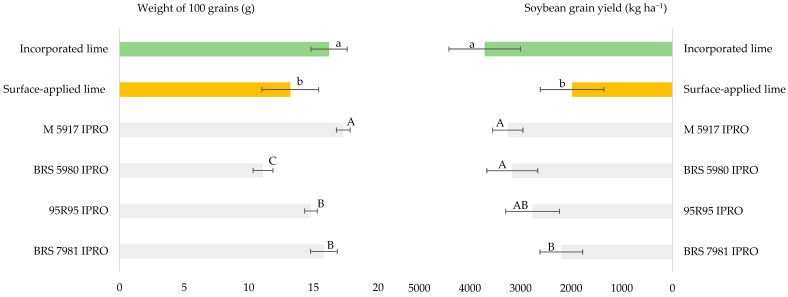
Weight of 100 grains and grain yield in an experiment comparing soil amendment placement in Campos dos Goytacazes, RJ, Brazil, during the 2021/2022 Season. Identical uppercase letters indicate statistically similar means between cultivars for the same variable, while lowercase letters indicate similarities between soil amendment placement, as determined by the Tukey test at a 5% probability level. For the strip-plot design, the coefficients of variation were as follows: for the weight of 100 grains, CV1 = 7.4%, CV2 = 8.0%, and CV3 = 6.0%; and for grain yield, CV1 = 21.2%, CV2 = 26.3%, and CV3 = 11.1%.

There was no interaction between cultivar and soil amendment placement for the 100-grain weight (Figure 2), and the highest weight was recorded in the area where lime and gypsum were incorporated into the soil (Figure 2). In contrast, the M 5917 IPRO cultivar had the highest grain weight, followed by BRS 7981 and 95R95 IPRO, which also differed from BRS 5980 IPRO (Figure 2). The soil amendment placement also influenced the components weight of 100 grains and soybean grain yield (Figure 2).

The evaluation of soybean grain yield indicated that the M 5917 IPRO and BRS 5981 IPRO cultivars achieved yields exceeding 3200 kg ha^−1^, outperforming the BRS 7981 IPRO cultivar, while the 95R95 IPRO cultivar had intermediate yields. The highest productivity was obtained in the treatment incorporating lime and gypsum, with a difference greater than 50%. There was no interaction between the soil amendment placement or cultivar (Figure 2).

## 3. Discussion

In this study, soil tillage for soybean crops was compared in two adjacent areas where lime and gypsum were applied either by incorporation to a depth of approximately 20 cm or through surface application. Because these soil amendment treatments were applied at the scale of two adjacent field areas, the results should be interpreted as a field-scale comparison between management strategies rather than as a fully replicated factorial experiment. Soil tillage was performed during the 2020/2021 growing season following the cultivation of different soybean cultivars. However, the evaluations for this study started from the following season, with the establishment of a new experiment. In the first year, crop grain yields were much lower than expected due to drought conditions, averaging around 1000 kg ha^−1^ in the area where lime and gypsum were surface-applied, and below 1500 kg ha^−1^ in the plots where these amendments were incorporated into the soil.

The soil was acidic, presenting high Al^3+^ content, and had a sandy texture. Evaluations were conducted following the conversion of the pasture to soybean crops, focusing on the effects of lime and gypsum application on the chemical characteristics of the soil and the development of different soybean cultivars.

The pH, Al^3+^, Ca^2+^, and Mg^2+^ analyses indicated that the amount of lime and gypsum applied in the previous crop (2020/2021) was insufficient to ensure high soybean yields, regardless of the soil amendment. The pH remained below the ideal level for plant development recommended for Brazilian soils, which is 6.0–6.8 in water [22]. The neutralization of Al^3+^, which is critical, was incomplete as levels of Al^3+^ ≥ 0.3 cmol_c_ dm^−3^ require the sum of Ca^2+^ and Mg^2+^ to reach at least 3 cmol_c_ dm^−3^ [23,24]. This value was not achieved, suggesting the need to revise the recommended lime and gypsum doses for the region, especially considering the diversity of the soil classes [4]. However, significant differences were observed between the two soil amendment strategies, indicating that the effect of soil pH on Al^3+^ availability may be more critical for soybean development than pH values alone.

When assessing the impact of surface soil amendment on soil fertility, soybean root development, and grain yield in a tropical region prone to drought, Bossolani et al. [25] concluded that lime, especially when combined with gypsum, improved soil fertility, as evidenced by increases in pH and levels of P, Ca^2+^ and Mg^2+^ throughout the soil profile. Carmeis Filho et al. [26] found that the classical liming recommendation based on the 0–20 cm layer is an underestimated approach for stable systems under no-tillage, with crop rotation and high input of crop residues throughout the agricultural year. This approach leads to an increased rate of lime application to increase the base saturation to 70%, as recommended for soybeans.

There was no significant interaction between soil amendment placements and sampling depth for pH, Al^3+^, Ca^2+^, and Mg^2+^, indicating that both soil amendment placements performed similarly. However, incorporating lime and gypsum yielded more pronounced results, increasing pH, Ca^2+^, and Mg^2+^ levels while reducing Al^3+^ across the soil profile. Thus, surface application was less effective at improving the chemical attributes of the soil during the study period. These results corroborated the findings reported by Rheinheimer et al. [27,28], highlighting the importance of incorporating lime to improve soil chemical conditions. The improvement in chemical attributes promoted by the incorporation of lime favored the deepening of the soybean root system, providing better conditions for deep root growth. This is crucial, especially during periods of water stress such as dry spells [29,30,31]. In fact, soil amendments can considerably increase drought tolerance by correcting soil acidity, increasing root growth, and improving nutrient availability [26,32].

In general, both P and K^+^ reached the 20–40 cm layer when lime and gypsum were incorporated, which did not occur with surface application (Table 1). The dynamics of P were influenced by precipitation with Al^3+^ in the soil, especially in the area with surface liming, and precipitation with Ca^2+^ in the top layer where lime and gypsum were concentrated [33]. Additionally, for both elements, it is possible that the greater growth of the root system, both from soybean in the first crop and Brachiaria used as a cover crop, contributed to transferring these elements to deeper soil layers.

Another important aspect to consider is the reduction in organic matter levels in all layers of the soil profile in the area using the incorporation amendment. This effect can be attributed to aggregate disruption by tillage, which exposes organic carbon and accelerates its decomposition [29] and also contributes to a decrease in CEC. This response is more commonly observed in sandy soils, whose CEC strongly depends on organic matter [34]. Furthermore, liming may initially enhance soil biological activity due to increased pH, stimulating organic matter mineralization and leading to short-term carbon losses. However, these effects are generally transient, as improved soil chemical conditions promote greater plant productivity and increased carbon inputs via crop residues and root biomass. Consequently, conservation management practices that enhance biomass production and organic matter return to the soil, such as no-tillage systems, may progressively offset these initial reductions and contribute to long-term soil carbon stabilization [35]. Importantly, the incorporation treatment involved soil tillage operations that may have influenced soil physical properties through mechanical loosening of the soil, potentially facilitating root penetration and exploration. Therefore, the results should be interpreted as the combined effect of soil amendment incorporation and soil mechanical disturbance rather than the isolated effect of amendment placement alone.

In summary, the evaluation of soil chemical attributes showed that incorporated lime and gypsum promoted an increase in pH and a reduction in exchangeable Al^3+^, and also increased the availability of Ca^2+^, Mg^2+^, P, and K^+^. There was also a reduction in Al^3+^saturation, an increase in the sum of bases, and an increase in base saturation, while organic matter slightly decreased. These results indicate that, under the conditions of this study, the incorporation of lime and gypsum through soil tillage improved soil chemical attributes compared with surface application in this pasture-to-cropland conversion system. They also underscored the importance of implementing management practices that increase organic matter levels over time, such as adopting no-tillage with year-round soil cover.

All cultivars showed adequate numbers of nodules, with cultivar M 5917 IPRO exhibiting the highest nodulation. This result was corroborated by Hungria et al. [36], who reported that a soybean plant with at least 15 nodules and 100 mg of dry nodule mass at the early flowering stage met the conditions necessary to satisfy its nitrogen requirements. Although BNF was not directly quantified, the adequate nodulation observed, the greater nitrogen accumulation in shoot biomass (approximately 30% higher in the area with incorporated lime and gypsum), and the satisfactory grain yield indicate that BNF was effective even in soils with low organic matter [10]. While cultivars differed in nodulation intensity and biomass accumulation, the beneficial effects of lime and gypsum incorporation were consistent across genotypes. Lime and gypsum application, alone or in combination, improved soil fertility and enhanced plant nodulation and nitrogen fixation in a tropical no-tillage intercropping system, thereby increasing the maize yield and plant nitrogen uptake [37].

The higher emission of secondary roots from soybean plants in the area by incorporating lime and gypsum indicated better soil chemical conditions, facilitating root exploration [14,38]. This condition was crucial for the absorption of water and nutrients, considering that the surface application of lime and gypsum did not neutralize the high Al^3+^ content, hindering root growth and aerial biomass [31]. The improvement of plant root development led to better overall plant growth, with a higher accumulation of Ca^2+^, Mg^2+^, K^+^, P, and N in the aerial parts. This effect has also been reported for soil amendments using lime and gypsum [37,38,39].

The lime and gypsum incorporation through tillage also influenced soybean production components such as the number of pods per plant and average grain weight. The incorporation soil amendment resulted in nearly twice the number of pods per plant with a higher grain yield by improving soil conditions, corroborating previous results [40].

Furthermore, it is important to note that the low rainfall recorded in January 2022 may have contributed to the reduction in grain yield [41], especially in the area with the surface application, where the number of secondary roots was the lowest. The observed difference in grain yield among soybean cultivars may be attributed to better adaptation to soil and climate conditions and interactions between soil amendment placement and local characteristics, such as latitude and altitude.

## 4. Materials and Methods

### 4.1. Local Area and Pre-Planting Procedures

This study was conducted during the 2021/2022 growing season (October to February) in an experimental area established during the 2020/2021 season at a private farm (Abadia) in the municipality of Campos dos Goytacazes, Rio de Janeiro State, Brazil (21°43′84″ S, 41°12′63″ W; 11 m a.s.l.). The area was previously cultivated with sugarcane for an extended period before being converted to pasture, predominantly *Brachiaria* spp., where it has remained for the past 10–15 years. Before treatment establishment, soil samples were collected using ten subsampling points across the experimental area at depths 0–20 cm and 20–40 cm and homogenized to obtain a composite sample for the initial soil characterization. The soil was classified as a typical dystrophic Fluvisol with a medium-sandy texture and the arable soil layer has a sandy texture [34]. The chemical and granulometric analyses [42] conducted before the experiment are shown in Table 4. The methods used for chemical characterization of the soil are mentioned further in Section 4.3.

The experiment followed a strip-plot design established in 2020, consisting of two main plots (approximately 70 × 70 m each). In May 2020, one main plot was plowed to approximately 20 cm, followed by broadcasting 1 t ha^−1^ of dolomitic lime and 0.5 t ha^−1^ of gypsum (CaSO_4_·2H_2_O), with subsequent incorporation to the same depth. In the second main plot, the same rates were applied without soil incorporation. Lime rates were calculated based on exchangeable Al^3+^ levels and crop Ca and Mg requirements based on regional recommendations in the Lime and Fertilization Manual for Rio de Janeiro State [24]. The lime contained 30% CaCO_3_ and 10% MgCO_3_, with an effective neutralizing value of 76%.

One month later, *Brachiaria ruziziensis* was sown in both main plots, followed by light disking to cover the seeds. At the beginning of October 2020, the grasses in both main plots were desiccated with glyphosate at a rate of 2 L ha^−1^ (product basis). After 10 days, different soybean genotypes were sown in both main plots to identify the crop adaptation to the region. The soybean seeds were inoculated with the recommended strains of *Bradyrhizobium* in a dose of approximately 1.2–1.5 million colony-forming units (CFU) per seed. Planting fertilization was a mixture of 100 kg ha^−1^ of P_2_O_5_ (appr. 45 kg ha^−1^ of P) and 80 kg ha^−1^ of K_2_O (appr. 65 kg ha^−1^ of K), using single superphosphate and potassium chloride as sources, along with 50 kg ha^−1^ of FTE BR-12 (containing approx. 3.0% S, 1.8% B, 0.8% Cu, 3.0% Fe, 2.0% Mn, 0.1% Mo, and 9.0% Zn). Soybeans were harvested in March 2021, leaving residues on the soil.

The data obtained from this initial genotype evaluation were not used in the present study, as the objective at that stage was solely to assess genotypic performance. Because the soil management treatments (incorporation versus surface application of lime and gypsum) were applied at the scale of large field strips, independent replication of this factor was limited. Therefore, the effects of soil management should be interpreted as a comparison between two field areas under contrasting management conditions rather than as a fully replicated experimental factor.

### 4.2. Experiment Set Up

For the evaluation in this study, an experiment was conducted during the 2021/2022 growing season, considering soil management in the previous season. This involved comparing two soil amendment placements, surface application and soil incorporation, which were carried out in 2020. In May 2021, *Brachiaria ruziziensis* was sown again in both management areas, followed by light disking to cover the seeds. At the beginning of October 2021, the grass was desiccated with glyphosate at a rate of 2 L ha^−1^. Subsequently, within each of the two management areas, four soybean cultivars were established in parallel strips approximately 3 m wide, with a row spacing of 0.50 m (six rows per strip). The cultivars were randomly arranged within each management area.

Before planting, the soybean seeds were inoculated with inoculants containing the two recommended *Bradyrhizobium* strains (a mix of strains SEMIA 5079 and BR 29). Approximately 6–7 doses of inoculants per hectare were used, providing a cell concentration of about 1.5 to 2 million CFU per soybean seed. Additionally, 100 mL ha^−1^ at concentration 10^8^ of *Azospirillum brasilense* inoculant (strains AbV-5 and AbV-6) was applied to the seeds, following the manufacturer’s recommendation. Planting fertilization was a mixture of 100 kg ha^−1^ of P_2_O_5_ (appr. 45 kg ha^−1^ of P) and 80 kg ha^−1^ of K_2_O (appr. 65 kg ha^−1^ of K), using single superphosphate and potassium chloride as sources, and 50 kg ha^−1^ of FTE BR-12 as a source of micronutrients.

The transgenic soybean cultivars BRS 5980 IPRO, BRS 7981 IPRO, 95R95 IPRO, and M5917 IPRO were cultivated under field conditions. The sowing density (seeds per hectare) was 240,000 for BRS 7981 IPRO, and 320,000 for the others, in accordance with the agronomic guidelines specific to each genotype. These differences in population density were maintained to reflect optimal management practices for each cultivar, as commonly adopted in commercial production. Sowing was performed under rain-fed conditions, starting with the first rain on 26 October 2021. The crop management followed the recommendations of EMBRAPA [43].

According to data from the National Institute of Meteorology (https://bdmep.inmet.gov.br accessed on 26 October 2024), the average maximum and minimum temperatures were 31.2 °C and 21.5 °C, resulting in a thermal amplitude of approximately 10 °C during the experimental period. The accumulated rainfall data, representing 10-day periods throughout the experiment, are shown in Table 5.

**Table 5:** Accumulated rainfall per decade (Instituto Nacional de Meteorologia (INMET), 2022) for the municipality of Campos dos Goytacazes-RJ.

Month	Days			
	1–10	11–20	21–30	
	Precipitation (mm)			Total
Oct	35	196	74	305
Nov	0	40	74	114
Dec	13	75	0	88
Jan	155	0	18	173
Feb	138	60	22	220
Mar	0	0	22	22
Total	341	371	210	922

### 4.3. Soil Samples and Chemical Analyses

Soil samples for chemical analyses were collected at depths of 0–10, 10–20, and 20–40 cm during the vegetative stage of the soybean crop and before the onset of flowering (approximately 40–45 days after sowing, R1 stage). Soil sampling was conducted only in the strip cultivated with BRS 5980 IPRO in both management areas in order to characterize soil chemical conditions associated with each soil amendment strategy. Along each strip, three sampling positions were established approximately 15–20 m apart. At each position, samples were collected both within the planting row (between plants in the second row) and in the inter-row space between the second and third rows. The average of row and inter-row samples was used to represent each sampling position. The levels of Ca^2+^, Mg^2+^, K^+^, P, pH, Al^3+^, potential acidity (H + Al), organic carbon, the sum of exchangeable bases (S), base saturation (V%), and aluminum saturation (m%) were determined.

Soil chemical analyses were performed following standard soil testing procedures: pH measured in water; Ca^2+^ and Mg^2+^ extracted using a saline solution and determined by atomic absorption spectrophotometry; K^+^ extracted with Mehlich-1 and quantified by flame photometry; P extracted with Mehlich-1 and determined by spectrophotometry; Al^3+^ extracted with saline solution and quantified by titration with NaOH; and soil organic matter estimated using the Walkley–Black method [42].

### 4.4. Per-Plant Evaluation Performance and Cultivar Grain Yield

To evaluate plant performance, five consecutive plants were sampled from the second row of each cultivar strip. Sampling was performed on the same day as soil collection. Five sampling positions were established along each strip, spaced approximately 15–20 m apart. Roots and shoots were separated and analyzed individually. By maintaining consistent sampling across cultivars (i.e., the same number of plants per plot), we ensured that comparisons were made at the individual plant level, to characterize cultivar-specific physiological responses under their respective recommended planting conditions. Nodules were detached from the roots, and the roots, nodules, and plant shoots were subsequently dried in a forced-air oven at 65 °C for 72 h. Chemical analyses were performed to estimate the nutrient content of the shoots following the methodology of Embrapa [42]. The nitrogen content was determined using a Vario Macro Cube C and N autoanalyzer (Elementar, Langenselbold, Germany) in the laboratory of EMBRAPA Agrobiology.

Approximately 70 days after planting (early R6 reproductive soybean growth stage), root samples were collected to evaluate root dry mass, following the previous sampling strategy with five plants and five replicates. The collection was performed using a flat spade, by making cuts 15 cm away from each side of the plants and removing the entire soil layer from 0 to 20–25 cm depth, ensuring that all roots within this profile were collected. Later, at harvest (R8 stage), five plants from each cultivar were sampled to determine the number of pods and the weight of 100 grains, with five replicates. Four samples were taken from each of the central rows in each plot, representing 3 m a length and a usable area of 3.0 m^2^ per sample, to determine grain yield (13% moisture).

### 4.5. Statistical Analysis

Statistical analyses were performed using R software version v 4.5.1 (R Core Team, Vienna, Austria; https://www.r-project.org). The study compared two field areas where lime and gypsum had previously been applied either by incorporation or by surface application. Within each management area, soybean cultivars were arranged in strips and evaluated using repeated sampling positions along each strip. Plant variables were analyzed considering soil management and cultivar as fixed factors, with sampling positions along the strips treated as repeated observations. Because soil management treatments were applied at the scale of two field areas, inference regarding this factor should be interpreted as a comparison between management areas under the conditions of this study rather than as a fully replicated experimental factor. Residual diagnostics were evaluated using the Shapiro–Wilk test for normality and Levene’s test for homogeneity of variances. When necessary, data were transformed to meet ANOVA assumptions. Soil chemical variables (pH, Ca^2+^, K^+^, P, and S) were transformed using √Y; nodule dry mass using √(Y + 100); root dry mass using log(Y); and root dry matter and number of secondary roots using the Box–Cox transformation. Statistical analyses were performed using transformed data, whereas the means presented in tables correspond to back-transformed values for clarity of interpretation. Treatment means were compared using Tukey’s test (*p* ≤ 0.05).

## 5. Conclusions

In summary, soybean performance improved more with the incorporation of lime and gypsum than with surface application. Incorporation increased soil pH, reduced Al^3+^ toxicity, and improved nutrient availability, which enhanced root development—particularly secondary root growth—and increased nodule biomass, both critical for effective biological nitrogen fixation and nitrogen accumulation. These benefits were consistent across cultivars, although differences were observed in nodulation, root biomass, and shoot biomass. Regardless of cultivar, the incorporation of lime and gypsum resulted in improved nodulation, enhanced nutrient uptake, a greater number of pods, higher grain weight, and ultimately increased yields.

It should be noted that, in the present study, the incorporation treatment involved soil tillage operations, which may have influenced soil physical conditions and organic matter dynamics. Therefore, the observed responses should be interpreted as the combined effect of soil amendment incorporation and soil mechanical disturbance rather than the isolated effect of amendment placement alone. In addition, lime and gypsum were applied together in all treatments, and thus the results reflect the combined effect of these amendments rather than their individual contributions.

From a practical standpoint, in degraded sandy soils characterized by subsoil acidity and low fertility the incorporation of lime and gypsum prior to crop establishment should be prioritized over surface application, especially in areas transitioning from long-term pasture to soybean cultivation. Surface application alone may be insufficient to adequately correct subsoil acidity under these conditions. However, because incorporation may reduce soil organic matter, this corrective practice should be combined with conservation-oriented strategies, such as no-tillage and the subsequent adoption of reduced soil disturbance, to ensure long-term soil sustainability.

## Figures and Tables

**Table 1:** Soil chemical characterization in an experiment comparing lime and gypsum application methods in soil cultivated with soybeans in Campos dos Goytacazes-RJ, 2021/2022 crop season.

Variable		Soil Amendment	Depth (cm)			Overall Mean	(CV) (%)
			0–10	10–20	20–40		
pH		Incorporated *	4.85	4.85	4.78	4.83 a	7.99
		Surface	4.27	4.38	4.26	4.30 a	2.28
		Average	4.56 A	4.62 A	4.52 A		1.93
Al^3+^	cmol_c_ dm^−3^	Incorporated *	0.49	0.44	0.53	0.49 a	42.57
		Surface	1.31	0.96	1.24	1.17 a	18.94
		Average	0.90 A	0.70 A	0.88 A		17.23
Ca^2+^		Incorporated *	2.13	0.84	0.68	1.22 a	11.38
		Surface	1.57	0.59	0.35	0.84 b	5.06
		Average	1.85 A	0.71 B	0.52 C		8.68
Mg^2+^		Incorporated *	0.68	0.42	0.30	0.46 a	14.86
		Surface	0.46	0.24	0.15	0.28 b	7.15
		Average	0.57 A	0.33 B	0.22 C		17.53
S		Incorporated *	3.14	1.34	1.06	1.84 a	3.09
		Surface	2.29	0.91	0.56	1.25 b	2.03
		Average	2.71 A	1.12 B	0.81 C		1.35
CEC		Incorporated *	5.89	5.87	5.86	5.87 b	13.75
		Surface	7.31	7.85	8.07	7.74 a	8.98
		Average	6.60 A	6.86 A	6.96 A		3.79
P	mg dm^−3^	Incorporated	23.39 Aa	6.79 Ba	7.23 Ba	12.47 a	5.83
		Surface	20.16 Ab	4.20 Bb	1.67 Cb	8.68 b	8.72
		Average	21.77 A	5.50 B	4.45 C		4.87
K^+^		Incorporated	128.19 Aa	31.35 Ba	30.85 Ba	63.46 a	7.19
		Surface	100.29 Ab	34.41 Ba	19.67 Cb	51.46 b	8.58
		Average	114.24 A	32.88 B	25.26 C		4.11
SOM	%	Incorporated	1.10 Ab	1.06 Ab	0.84 Bb	1.00 b	9.53
		Surface	1.68 Aa	1.38 Ba	1.30 Ba	1.4 a	6.28
		Average	1.39 A	1.22 B	1.07 C		3.59
V		Incorporated	53.76 Aa	23.11 Ba	18.03 Ca	31.63 a	10.83
		Surface	31.83 Ab	11.90 Bb	6.99 Cb	16.91 b	8.50
		Average	42.80 A	17.50 B	12.51 C		5.62
m		Incorporated *	12.67	22.11	28.91	21.23 b	4.14
		Surface	33.94	45.97	64.28	48.06 a	14.51
		Average	23.30 C	34.04 B	46.59 A		12.31

S—exchangeable bases; V%—base saturation; m%—Al^3+^ saturation; CEC—cation exchange capacity. Means followed by different uppercase letters within each column are significantly different among soil depths, and means followed by different lowercase letters within each row are significantly different between soil amendment placement, according to Tukey’s test (*p* ≤ 0.05). Means without letters indicate that differences were not significant (*p* > 0.05). For variables marked with (*), no significant interaction between soil amendment and soil depth was observed. Data for pH, Ca^2+^, K^+^, P, and S were square-root-transformed for statistical analysis; original means are presented.

**Table 2:** Nodule dry mass, nodule number, primary root length, secondary root number, root dry mass, and shoot dry matter of soybean in an experiment comparing soil amendment placement in Campos dos Goytacazes, RJ, Brazil, during the 2021/2022 growing season.

Cultivar	Nodules Dry Mass (mg Plant^−1^)	Number of Nodules of (Planta^−1^)		Primary Root Length (cm)			Root Dry Mass (g Plant^−1^)		
BRS 5980 IPRO	304.4 A	45.0 A		19.25			3.19 A		
BRS 7981 IPRO	253.6 AB	29.8 B		18.40			1.60 B		
M 5917 IPRO	160.0 C	33.5 B		18.85			2.72 A		
95R95 IPRO	207.4 BC	27.6 B		17.40			1.70 B		
CV (%)	10.3	24.7		10.4			16.0		
Soil amendment									
Incorporated	264.4 A	33 A		18.4			2.34 A		
Surface	198.2 B	34 A		18.5			2.26 A		
CV (%)	15.1	19.9		9.9			14.7		
Cultivar	Number of secondary roots			Shoot dry matter (g plant^−1^)					
	Incorporated	Surface	Average	Incorporated		Surface		Average	
BRS 5980 IPRO	61.2	41.2	51.2 B	11.64 Ba		10.24 ABb		10.94 B	
BRS 7981 IPRO	68.0	63.0	65.5 A	10.47 Ca		10.71 Aa		10.50 BC	
M 5917 IPRO	66.0	50.2	58.1 AB	13.24 Aa		10.50 Ab		11.87 A	
95R95 IPRO	66.0	54.2	60.1 AB	10.72 BCa		9.40 Bb		10.06 C	
Average	65.3 a	52.1 b		11.51 a		10.21 b			
CV (%)	26.7	13.6	24.1	2.2		1.8		2.4	

Means followed by different uppercase letters within each column are significantly different among cultivars and means followed by different lowercase letters within each row are significantly different between soil amendment placement, according to Tukey’s test (*p* ≤ 0.05). Means without letters indicate that differences were not statistically significant (*p* > 0.05). Data for nodule dry mass were square-root-transformed [√(Y + 100)]; root dry mass was log-transformed [log(Y)]; and root dry matter and number of secondary roots were transformed using the Box–Cox procedure for statistical analysis.

**Table 3:** Calcium, magnesium, potassium, phosphorus, and nitrogen accumulation in the soybean shoot in an experiment comparing soil amendment placement in Campos dos Goytacazes-RJ, 2021/2022 crop season.

Cultivar	Ca^2+^ (mg Plant^−1^)			CV (%)
	Incorporated	Surface	Average	
M 5917 IPRO	148.9 A	98.8 A	123.8 A	
BRS 5980 IPRO	125.4 B	92.8 AB	109.1 B	
95R95 IPRO	114.2 B	82.4 B	98.3 C	
BRS 7981 IPRO	93.9 C	81.9 B	87.9 D	
Average	120.6 a	88.9 b		9.5
CV (%)	6.7	9.9		
Cultivar	Mg^2+^ (mg plant^−1^)			CV (%)
	Incorporated	Surface	Average	
M 5917 IPRO	49.1 A	32.1 A	40.6 A	
BRS 5980 IPRO	38.7 B	32.5 A	35.6 A	
95R95 IPRO	33.7 B	25.7 B	27.9 B	
BRS 7981 IPRO	28.0 C	27.8 AB	29.7 B	
Average	37.4 a	29.5 b		
CV (%)	8.4	5.0		9.5
Cultivar	K^+^ (mg plant^−1^)			CV (%)
	Incorporated	Surface	Average	
M 5917 IPRO	353.9 A	240.7 AB	297.3 A	
BRS 5980 IPRO	300.8 B	255.3 A	278.1 A	
95R95 IPRO	249.5 C	201.2 B	225.4 B	
BRS 7981 IPRO	211.3 C	197.5 B	204.4 B	
Average	278.9 a	223.7 b		
CV (%)	10.6	8.82		10.7
Cultivar	P (mg plant^−1^)			CV (%)
	Incorporated	Surface	Average	
M 5917 IPRO	46.6 A	31.9 A	39.25 A	
BRS 5980 IPRO	36.7 AB	33.2 A	34.95 A	
95R95 IPRO	37.4 AB	29.8 A	33.6 A	
BRS 7981 IPRO	29.2 B	34.3 A	31.5 A	
Average	37.5 a	32.3 b		
CV (%)	23.9	5.2		17.4
Cultivar	N (mg plant^−1^)			CV (%)
	Incorporated	Surface	Average	
M 5917 IPRO	418	296	358.2 A	
BRS 5980 IPRO	304	244	293.6 BC	
95R95 IPRO	350	242	273.7 B	
BRS 7981 IPRO	264	222	243.4 C	
Average	333.2 a	251.3 b		
CV (%)	8.0	7.5		11.1

Means followed by different uppercase letters within each column are significantly different among cultivars and means followed by different lowercase letters within each row are significantly different between soil amendment placement, according to Tukey’s test (*p* ≤ 0.05). Means without letters indicate that differences were not statistically significant (*p* > 0.05).

**Table 4:** Chemical and granulometric characterization and soil particle size distribution before the lime and gypsum application in the 2020/2021 season in Campos dos Goytacazes-RJ.

Depth (cm)	P	K	Ca	Mg	Na	Al		H + Al	pH
	---mg dm^−3^---		----------cmol_c_ dm^−3^---------						
0–20	5	43	1.5	0.6	0.08	0.5		3.1	4.6
20–40	4	23.5	1.1	0.4	0.07	1		4.8	4.6
Depth (cm)	SOM	S	CEC	V	m	Cu	Fe	Mn	Zn
	%	---cmol_c_ dm^−3^---		-----%-----		---------mg dm^−3^---------			
0–20	1.74	2.3	5.47	43	18	1.01	189.5	1.39	14
20–40	1.17	1.65	6.6	26	37	1.02	194.8	051	5.22
Depth (cm)	Particle size distribution (%)								
	Sand			Silt				Clay	
0–20	75			13				12	
20–40	73			8				19	

## Data Availability

Data are available from the corresponding author upon reasonable request. The data are not publicly available due to University Thesis rules.
